# Supplementary material for: A Humanized Yeast Phenomic Model of Deoxycytidine Kinase to Predict Genetic Buffering of Nucleoside Analog Cytotoxicity
Source: Genes (Basel). 2019 Sep 30;10(10):770. doi: 10.3390/genes10100770 (PMC6826991; doi:10.3390/genes10100770)
Supplement: Supplementary file 1 [file genes-10-00770-s001.zip › Additional_Files/Additional_File7_GOTermHeatmaps.zip!/Additional_File7_GOTermHeatmaps/positive_regulation_of_filamentous_growth_of_a_population_of_unicellular_organisms_in_response_to_starvation.pdf]

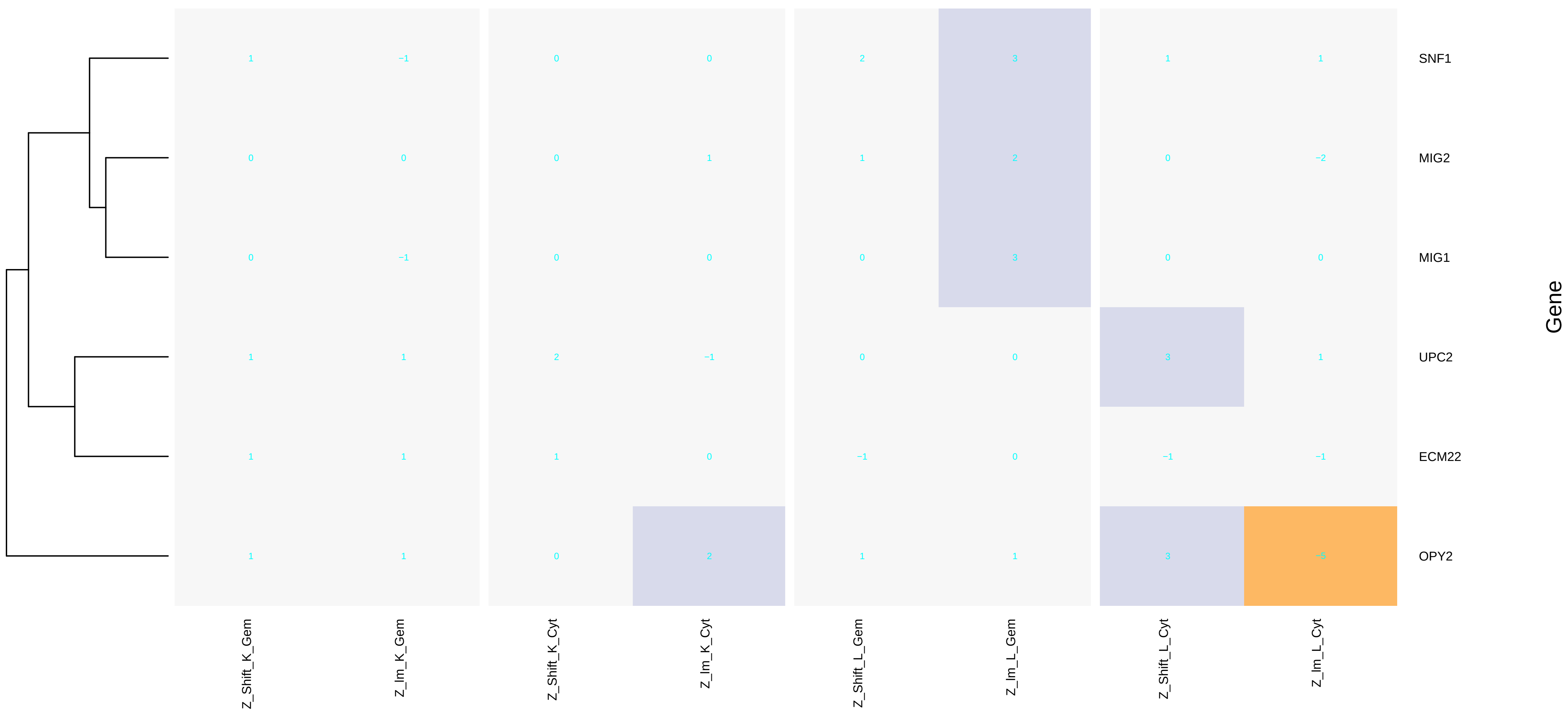

Gene
SNF1
MIG2
MIG1
UPC2
ECM22
OPY2
Z_Shift_K_Gem
Z_Im_K_Gem
Z_Shift_K_Cyt
Z_Im_K_Cyt
Z_Shift_L_Gem
Z_Im_L_Gem
Z_Shift_L_Cyt
Z_Im_L_Cyt
1 −1 0 0 2 3 1 1
0 0 0 1 1 2 0 −2
0 −1 0 0 0 3 0 0
1 1 2 −1 0 0 3 1
1 1 1 0 −1 0 −1 −1
1 1 0 2 1 1 3 −5
